# Gateway Effects: Why the Cited Evidence Does Not Support Their Existence for Low-Risk Tobacco Products (and What Evidence Would)

**DOI:** 10.3390/ijerph120505439

**Published:** 2015-05-21

**Authors:** Carl V. Phillips

**Affiliations:** The Consumer Advocates for Smoke-Free Alternatives Association (CASAA), P.O. Box 652, Wilbraham, MA 01095-0652, USA; E-Mail: cphillips@casaa.org; Tel.: +1-202-241-9117

**Keywords:** gateway hypothesis, tobacco harm reduction, e-cigarettes, smokeless tobacco, causation, testimonial evidence, hypothesis testing, cross-sectional data analysis, scientific inference

## Abstract

It is often claimed that low-risk drugs still create harm because of “gateway effects”, in which they cause the use of a high-risk alternative. Such claims are popular among opponents of tobacco harm reduction, claiming that low-risk tobacco products (e.g., e-cigarettes, smokeless tobacco) cause people to start smoking, sometimes backed by empirical studies that ostensibly support the claim. However, these studies consistently ignore the obvious alternative causal pathways, particularly that observed associations might represent causation in the opposite direction (smoking causes people to seek low-risk alternatives) or confounding (the same individual characteristics increase the chance of using any tobacco product). Due to these complications, any useful analysis must deal with simultaneity and confounding by common cause. In practice, existing analyses seem almost as if they were designed to provide teaching examples about drawing simplistic and unsupported causal conclusions from observed associations. The present analysis examines what evidence and research strategies would be needed to empirically detect such a gateway effect, if there were one, explaining key methodological concepts including causation and confounding, examining the logic of the claim, identifying potentially useful data, and debunking common fallacies on both sides of the argument, as well as presenting an extended example of proper empirical testing. The analysis demonstrates that none of the empirical studies to date that are purported to show a gateway effect from tobacco harm reduction products actually does so. The observations and approaches can be generalized to other cases where observed association of individual characteristics in cross-sectional data could result from any of several causal relationships.

## 1. Introduction

An often-claimed downside of harm reduction strategies is that the lower-risk alternative to the high-risk behavior is a “gateway” to the high-risk behavior. That is, the availability or promotion of the low-risk option causes some people who would not otherwise have done so to adopt the high-risk behavior. Various studies have purported to show that such effects exist but, at least for the case of tobacco, none of the evidence actually supports the claim. Rather, they demonstrate the faulty understanding of the authors about what evidence would support the claim. 

Most of the analysis presented here applies to any gateway claim, but the presentation focuses on tobacco harm reduction (THR) products, low-risk alternatives to smoking, including smokeless tobacco and e-cigarettes. The analysis also generalizes to any situation where cross-sectional data is used to test a particular causal claim when competing causal pathways appear to be much stronger causes of the association. In the THR case, there is clear confounding due to a common cause, which can be thought of as the generic unspecified common cause, typically designated “U” in the methods literature, or specifically in terms of a propensity toward liking the effects of tobacco/nicotine and being willing to resist the social pressures that demand abstinence. There is also clear causation in the opposite direction of the gateway: low-risk tobacco products, especially e-cigarettes, are almost exclusively used as a substitute for smoking, causing an association between their use and the use of cigarettes. Any methodology attempting to identify the effects of one causal pathway among those three—especially if, as with the THR gateway claim, the pathway of interest undoubtedly contributes less to the association than the others—requires a great deal of thought about how to use data.

Gateway claims became common starting in the 1950s, as a U.S. Drug War tactic aimed at demonizing cannabis use. (The term itself appears to have been coined in the 1970s as part of that effort and of the still ongoing studies by one researcher who does not appear to have seriously considered the complications presented in the present paper [1,2].) The biological and social effects of typical patterns of cannabis use are inadequate to justify a prohibitionist war, and there was even less evidence suggesting it was harmful at that time. So the claim was adopted that it is seriously harmful because it causes some users to “move on” to “hard drugs”. A similar tactic has been adopted by opponents of THR strategies, in which they claim that their use causes substantial harm because it causes some people to start smoking (e.g., [3,4,5,6,7]). It has even been claimed by the coiner of the term “gateway” that e-cigarettes are a gateway to cocaine [2], though that claim is easily debunked [8].

Gateway claims can serve as a refuge for activists who profess concern about health or welfare, but really oppose a particular behavior for some other reason and cannot find any health- or welfare-based objection. The claims serve as a rationalization that is sufficiently ominous to influence the thinking of an uncritical target audience due to the common tendency to fixate on the consequences of a possible negative outcome, ignoring its probability and thus net expected value. The consistent lack of quantification in the claims (*i.e.*, *how many* gateway cases have occurred or are predicted) tends to suggest that their only goal is to trigger this cognitive bias.

Opponents of THR invoke the gateway claim in discussions about eliminating the products entirely (which would stop any gateway effect), but also about regulating where the products can be used (which would have no apparent effect on any gateway). They invoke the same claims when the discussion is about whether to encourage the use of low-risk alternatives among current smokers (which could not possibly create a gateway). This suggests the gateway is, to them, merely a rhetorical tactic, not a genuine concern. Indeed, a leading anti-THR strategy is to claim that the low-risk products are much higher risk than they are, which, if believed, tends to *increase*, rather than decrease, any gateway effect because it tells anyone using those products that they might as well smoke [9]. It has recently been argued that propaganda campaigns against e-cigarettes have caused many vapers (users of e-cigarettes) to switch back to smoking [10,11]. These observations suggest that the stated worry about gateway effects is disingenuous.

But even if the gateway claim is primarily used as a rationalization for other goals, there is a legitimate scientific question about the extent to which it occurs. There are certainly policy-makers and other observers who have been persuaded to believe there are substantial effects that should motivate policy. Thus, there is value in analyzing what empirical evidence is potentially useful in assessing whether such effects exist. Even if the conclusion is that there is little chance of demonstrating an effect, there is value in identifying what research could (and thus what research could not) detect it, because it serves to point out which claims are groundless.

The following analysis explains why most of the evidence that is typically cited in support of gateway claims is invalid, and is perfectly consistent with there being no gateway effect or even with extreme alternative hypotheses. The empirically-based claims that are made only support the hypothesis that people who use one tobacco product are more likely than the average person to use another. They do not support a gateway claim over two other candidate causes of such an association. However, the present analysis also points out that some of the arguments most frequently made against gateway claims are also faulty. It concludes with an assessment of what empirical evidence could be used to support or refute gateway claims and an example of applying such reasoning.

While some of the particulars of this analysis are rather unusual, there are many general lessons about analyzing cross-sectional data. The most obvious is about not confusing causation in one direction with confounding or causation in the other direction, a straightforward point but one that can always benefit from additional teaching examples. More subtle potentially generalizable lessons can be found in the proposed strategies for dealing with that challenge, in particular how to devise severe hypothesis tests using data that is not as rich as we might prefer.

## 2. Analysis

### 2.1. The Logic of the Gateway Claim

Describing the mechanism by which a cause might lead to an effect is frequently useful. Cause-effect relationships are often established without knowledge of a mechanism when studying the complexities of human biology. But in a relatively straightforward process of preferences, decisions, and volition, as in the present case, it is possible to characterize the necessary mechanism. Doing so offers some clues about useful empirical research while also emphasizing that this is an extraordinary claim that calls for extraordinary evidence. 

Gateways certainly can exist. For example, medical opiates appear to sometimes be a gateway to heroin, with some people who never would have considered using heroin acquiring a strong preference for consuming opiates due to medical usage (which is often labeled addiction or dependence, though such characterizations are immaterial for present purposes), and then discovering that the net benefits of heroin are even greater, considering both the effectiveness and the relative cost of obtaining it. But for tobacco products there is no analogous story. The barriers to starting smoking are quite low, in contrast with heroin use, so there are not formidable obstacles that require a barrier-lowering experience or “ramping up” effect. Moreover, while some THR alternatives are a bit more difficult to acquire than cigarettes in some places due to bans or anti-THR taxes, they rarely become unavailable to a user who became accustomed to them as can happen with medical opiates. Thus, the most obvious real gateway problem that currently exists has little resemblance to use of tobacco products.

Consider the scenario that is needed to create a gateway case of smoking: Someone chooses abstinence over smoking, though the latter is an easy option. Assume that this individual is among the approximately half of the population that benefits from using tobacco (and thus is even a candidate to start using the products). The choice of abstinence shows she is among the approximately half of that subpopulation who decide that the costs of smoking outweigh the benefits (which is why she does not smoke in spite of those benefits). She is then exposed to a low-risk alternative that she prefers to abstinence. Why, exactly, would she then switch from this product to what had been her least-preferred option, smoking? The logic of this is difficult to conceive and is never explained by those who claim there is such a gateway effect. 

It is not impossible, of course. At the moment, smoking delivers nicotine more effectively than existing low-risk alternatives, and includes other psychoactive agents that are not present in extracted nicotine products such as e-cigarettes. It is thus possible that someone who never even tried smoking because of concerns about its health effects could discover how much she would like it by discovering she likes the low-risk alternative. This could *conceivably* lead her to conclude that smoking has benefits that are greater than its costs. It is also possible that she had tried smoking and was not enthralled, but acquires a growing taste for nicotine via the use of a low-risk alternative which then makes the better delivery more attractive than it was initially. However, in both cases, the new *net marginal* appeal of smoking would have to be positive. That is, the advantages of smoking over and above those already provided by the low-risk product would have to be greater than the health costs of smoking (net of the trivial health costs of the alternative product), and enough to get her to overcome the inertia to switch products. Even if the change in total appeal promoted smoking over abstinence, a much greater change would be required to elevate it over the low-risk product. It is difficult to imagine this scenario occurring.

It is significant that proponents of the gateway claim never even attempt to present a scenario for why someone would ever make the gateway switch. This is presumably because presenting this scenario would illustrate that our prior beliefs should be that the effect is improbable and rare.

It is possible to *engineer* a gateway from low-risk tobacco products to smoking via public policy, similar to the opiates story. A spike in the punitive smokeless tobacco taxes in Canada caused some nonsmoking smokeless tobacco users to switch to cigarettes, at least occasionally, because they became much cheaper than the smokeless product they preferred [12]. A ban (either literal or *de facto*) on e-cigarettes, which is still possible in many jurisdictions, could drive some non-smokers to smoke, though almost all would be former smokers who resume smoking, so it is not clear this should be called a gateway. Thus an incentive can be created to switch from a low-risk tobacco product to smoking, but little such incentive exists naturally.

### 2.2. Causation

As is common when terms from political rhetoric find their way into scientific analysis, the word “gateway” is seldom defined by those who use it. For the gateway claim to match the policy discussion that surrounds it, it must be interpreted as the low-risk product *causing* the use of the high-risk product. This is sometimes stated explicitly and can be inferred in other cases. (The claim that the high-risk behavior is caused to *persist* as a result of the low-risk alternative, but not caused to initiate, has similar implications to the gateway claim. But it is a fundamentally different phenomenon, requiring different research, and so is not included here and does not seem to be properly included in the “gateway” label.)

An alternative potential meaning, “C (the ostensible cause) merely precedes E (the ostensible effect)” is clearly not what is meant, though there is sometimes a bait-and-switch use of such a definition. “C is a convenient step on the way to E, which is the ultimate goal” is the literal interpretation of the metaphor (you pass through a portal because you are trying to arrive at something on the other side of it, so the destination causes the use of the gateway, not the other way around), but clearly this is not the intended interpretation. Similarly, sometimes THR proponents attempt to hijack the term, saying “yes, there is a gateway, but only *away from* smoking.” While it is easy to understand the temptation, this is not a legitimate argument; the word has an accepted meaning and trying to redefine it does not constitute an argument that the phenomenon does not exist.

Though the philosophical nuances of the concept of causation are extensively debated, for practical purposes the statement “C causes E” is defined by the counterfactual statement, “if C is true then E will occur and if C is not true then E will not occur, holding constant everything else that is not caused by C.” Granted the last condition, as phrased, is a bit circular (technically it is an infinite recursion), but it gets the point across for practical purposes. *Counterfactual* refers to the fact that if one of those if-then scenarios is observed then the other cannot be, and so is necessarily counter to the facts. This is why causation can never be observed or proven, only inferred.

The previous paragraph describes causation at the level of a single event or individual. At the population level, a cause is a factor that for *some* individuals has the properties of C in the definition. When causation is inferred from data about a population (as it almost always is in social science), it will not be known which particular cases of E were caused by C and which were not (unless C is a necessary cause—see below). That C is causing some cases of E can be inferred from particular patterns in data. However, as explained below, the simplest observation about the data—a correlation between the variables that are candidates for C and E—is not in itself sufficient information, and further analysis is required before the claim is established.

Every worldly outcome has multiple causes. Someone becoming a smoker on a particular day might be caused by a friend handing her a first cigarette, but it is also caused by her living long enough to become a smoker and by the historical fact that the proto-tobacco plant evolved and was discovered and cultivated by humans. Practical discussion tend to only address causes like the first, the one that might *not* be a cause of her ever becoming a smoker. If the world were changed such that tobacco did not exist, she would never become a smoker. However, if the world were changed so she was home sick instead of experiencing the particular friend event, she might not have become a smoker that day, but some other event in her life could have caused her to become a smoker.

The friend event is the only one on that list that is not a *necessary* cause of her ever becoming a smoker. A necessary cause is anything whose absence would ensure that the outcome would not occur no matter what else was true. Put another way, if you listed every set of causes that would lead to the outcome if all in the set were true, a necessary cause would appear in every such list. In social sciences, necessary causes are seldom very interesting, though the failure to understand the concept sometimes creates confusion. 

A *sufficient* cause is a cause that produces the effect no matter what else is true. So if we created the list of each combination of causes that result in an outcome, one of the entries on the list is the sufficient cause by itself. Single sufficient causes do not exist in the real world, except when they are tautological, definitional, or a constructed conjunction. Smoking your 100th cigarette causes you to become an ever-smoker if “ever-smoker” is defined as having smoked at least 100 cigarettes. It is always possible to take any list of causes that together are sufficient and combine them into one long conjunction and call it *a* cause. Indeed, in epidemiology that conjunction is often described as a sufficient cause, following the terminology of Rothman’s “sufficient-component cause model”. But familiarity with this convention can distract from the fact that what we think of as a *single* event (not a conjunction) is never a sufficient cause, and failure to understand the difference between sufficient cause and cause creates confusion. For example, it is sometimes suggested that since most smokers who have tried e-cigarettes are still smokers, it must be that e-cigarettes must not cause smoking cessation. This is wrong because it merely shows that trying an e-cigarette is not *sufficient* for smoking cessation; it is still a cause (for some individuals, and thus at the population level).

A critical observation is that many effects are *overdetermined*, meaning that all the causes of two or more of the sets in the list are met. This means that if you changed the world so that one set of causes were no longer all true, the outcome would still be caused by another set if it remained unchanged. In the above example, if the subject, who we know has an inclination to smoke, was not handed her first cigarette by a particular friend on a particular day, there is still a good chance that she would become a smoker because some other set would be completed (e.g., a month later she asked for a puff from a different friend). In common language, each item on each redundant set of potential causes for an overdetermined effect is often called a cause, though the counterfactual definition says that *none* of them (except those that appear in every list like “tobacco exists”) are causes for the outcome ever occurring because it still would have occurred in their absence.

The reader may have noticed the seemingly tortured phrasing distinguishing causes of “being a smoker” or other conditions *ever* coming into being—sometime and somehow—from causes of specific event occurring at a specific time. This is a critical distinction. Even if e-cigarette use caused the particular chain of events that led to someone taking up smoking, it might not have caused him to *be a smoker*. If he would have started smoking in the absence of e-cigarettes due to some other prompt, then the outcome “being a smoker” was overdetermined and so e-cigarettes did not cause that outcome, and thus were not a gateway. Note the relationship to a common misunderstanding about epidemiologic statements like “smoking causes millions of deaths”: Some observers balk at such claims, protesting that those individuals would have died whether or not they smoked. But the unstated caveat in the epidemiologic claim is “caused deaths to occur *at a particular time that would not have otherwise occurred at that time*.” The confusion is created by using phrasing that in common language could mean either that or “caused deaths that would have never occurred otherwise”.

This is not a philosophical or semantic technicality; it is crucial to understanding the gateway claim and how to test it. A meaningful gateway claim is a “would have never occurred otherwise” claim, not an “at that time” claim. If that person would have become a smoker in a world without e-cigarettes, then e-cigarette use cannot be blamed for the ultimate outcome even if it was the trigger for the particular path he followed. Of course, it may be useful to study the very different question “what are people’s proximate triggers for taking up smoking?”, apart from whether they are actually the causes of them becoming a smoker. But since the practical gateway claims are about an exposure creating smokers from would-be nonsmokers, the technical logical definition clarifies what is really being claimed. 

#### Confounding and Reverse Causation

Confounding, because of the way it is presented in the context of statistical analyses, is sometimes not understood to be a statement about causation. The association between C and E in a population is confounded if there is a difference in the rate of E between the subpopulation who have C and subpopulation who do not have C *that is not caused by C*. That is, if you could change the world such that no one had C, but all else was the same, the rate of E would still be different between the two subpopulations. A simplistic comparison of the groups, then, would attribute this difference to C even though it was not caused by C. 

Notice that the definition of confounding does not make reference to the concept of *confounders*, which refers to either other variables that might be the causes of that difference or, more typically, to other variables that could be put into a statistical analysis that might allow you to sort out the effect of C from the confounding. These are quite different concepts, and the latter—available variables that help control for confounding—are better labeled *deconfounders* [13]. A true confounder would include some characteristic U that is a cause of E and is also a cause of C. There are other more complicated causal roles true confounders also play, but this phenomenon—a *common cause*—creates the greatest challenge when exploring gateway claims. Liking the effects of nicotine, for example, causes the use of cigarettes and the use of smoke-free alternatives.

It might be possible to measure U and thus use the measurement as a deconfounder variable, or to measure proxies for U and use them as deconfounders. In practice in public health research, “controlling for confounding” consists of throwing in whatever variables the researcher has that might be (though often probably are not) proxies for real confounders. While methods have been developed in epidemiology to improve the choices of deconfounder variables (e.g., causal flowcharts), they are almost never employed. Many covariates included in epidemiology models seem about as likely to “adjust” the measure of effect further from the true value as to adjust it closer. For example, the habit of controlling for race, because the variable happens to be in the dataset, when there is no reason to believe that race confounds or modifies the ostensible causes of interest, is functionally equivalent to controlling for a column of random numbers. Even worse, controlling for intermediate factors on a causal pathway (e.g., for blood pressure when estimating the effects of smoking on cardiovascular disease) will tend to mask the real effect.

But even when researchers are skilled at identifying optimal deconfounders, it is often the case that either no available variables offer good proxies for the postulated confounders or that they are not measured accurately enough for statistical adjustment to occur. For example, a researcher trying to assess whether an exposure causes smoking may realize that he needs to control for propensity for depression and willingness to defy social pressure, but the available covariates (age, race, level of education, income, *etc.*) offer little deconfounding for these. Controlling for the heterogeneous preferences for nicotine seems nearly impossible. Seldom do researchers even discuss what hypothesized confounder variables they are attempting to proxy for, let alone offer any argument that the control variables they threw into their model achieve the goal of deconfounding.

Another causal pathway that has similar implications to confounding is *reverse causation*. That is, E is actually causing C. Association of two variables always works in both directions: if E is associated with C, then C is associated with E. Thus, some additional analysis, beyond observing a correlation in the data, is needed to draw conclusions about which direction the causation flows, even in the absence of confounding. For many areas of research, the reverse pathway is so implausible that it may be valid to completely ignore it, but for some questions—particularly including analysis of constellations of behavioral choices—this is not so. (*Association* is most easily interpreted as “knowing the value of one variable for an individual affects your probability of the other”. *Correlation* refers to the same phenomenon, but is a mathematical property of population data rather than a reference to worldly phenomena that is meaningful at the individual level. Note also that associations or correlations can be positive (C true increases the likelihood of E true) or negative (decreases the likelihood), but when used without clarification the words generally refer to the positive.)

The logic of the gateway claim shows it to be an extraordinary claim, while alternative explanations inevitably create an association. Still, co-occurrence of all three of those causal pathways is possible, and thus there could also be a gateway effect, but further evidence is needed to affirmatively support such a claim. 

### 2.3. Use of Similar Drugs is Inevitably Associated

Give the above observations, it is impossible to imagine that use of low-risk tobacco products would not be associated with smoking. Still, there is value in further exposition to clarify the thinking of serious researchers and to push back against those who implicitly deny the obvious.

Someone who smokes a particular pack of Marlboros will almost certainly smoke another pack of Marlboros, though obviously the fact that the particular first pack was shipped to his local store and he bought it did not cause him to smoke the other. He is also far more likely than the average person to smoke or have smoked a pack of another brand of cigarettes. The probability drops somewhat when the other brand is menthol; the closer the similarity of the products, the stronger is the association of their use. The association is smaller, but still far from null for non-cigarette tobacco products. We would also predict a greater probability of smoking cannabis or using cocaine than would be average for someone of the particular age, cohort, and demographics, since he has demonstrated a greater desire and willingness to use drugs compared to the average person. It should be obvious that none of those other higher probabilities were caused by the fact that the particular pack of Marlboros in question found its way to that consumer’s corner store.

This is a cartoon example, and yet, blaming that particular pack for the other behaviors is exactly the logic of most empirically-based gateway claims. Common gateway claims are based on the simple observation that a larger proportion of those who smoke have used a low-risk alternative as compared to those who do not smoke.

For example, Haddock *et al.* [14] claimed to demonstrate a gateway effect by observing a mere association between smokeless tobacco use and smoking. They also reported a strong association with other risk-taking or socially disparaged behaviors (e.g., motorcycle riding) that were obviously not caused by smokeless tobacco use, but were apparently unaware that this illustrated the fallacy of their conclusion. More recently, Glantz *et al.* have claimed that e-cigarettes are causing teenagers to smoke, when their data [15,16] actually provided no support for that claim and instead better supported the claim that those who already smoked were seeking a substitute [17]. 

### 2.4. Precedence Has Epistemic Value, but It Is Generally Overstated 

A variation on the claim that mere association indicates a gateway is to suggest that the association must be causal if use of the low-risk product preceded the use of the high-risk product. Some gateway claims are based on merely showing that some individuals used the low-risk product before becoming smokers, and then discussing the result as if it supported the much stronger claim of a gateway. This is clearly invalid, not just because of the change in definition, but because there is no reason to care about non-causal precedence. The classic example is that almost every heroin user consumed milk before ever touching heroin. No one who is not trying to mislead uses the term gateway to refer to mere precedence. 

A slightly more informative approach is to combine association and precedence, observing that nonsmokers who use or have tried a low-risk alternative are more likely to later become smokers than similar nonsmokers who have never used tobacco products. But while this substantially reduces (but does not eliminate) the problem of reverse causation, it offers minimal reduction in confounding and so remains inadequate evidence for a gateway.

However, contrary to a large portion of criticisms of gateway claims, the lack of the “right” temporal ordering does not disprove the gateway claim. That is, just because someone tried a cigarette before ever using the low-risk alternative does not mean that the low-risk alternative is not the cause of later smoking. Trialing smoking in the past, or even a period of regular smoking followed by cessation, does not immunize someone against the possibility of another product causing later smoking. Causation of someone trying his first cigarette could be ruled out by the simplest measures of temporality, but that is not the outcome of interest. Temporality data is not useless; it can be used to identify individuals who are much more likely to be candidates for the gateway, as is done below. But simply declaring that someone who used a cigarette first cannot be a gateway case is incorrect.

### 2.5. Testimony is Critical, though Overdetermination Complicates Inference

A good method for assessing causation is to ask subjects what they perceive to be a cause. This is not definitive, for obvious reasons, but is potentially among the most useful data when it is an option (*i.e*., the objects of study are people and the apparent link between cause and effect is easily observable). It is a neglected research option in epidemiology, which seems largely to result from researchers’ failure to acknowledge that they are engaged in social science rather than clinical research [18], although obvious political motivations are often the explanation for ignoring the available testimonial evidence (e.g., [19]). An individual’s testimony about what caused his cancer is worthless because there is no way anyone can know that. But his testimony about why he changed his behavior is useful scientific evidence.

However, even if someone (accurately) communicates a proximate cause of smoking such as “my friends and I were using e-cigarettes all the time, and I wanted to try the real thing”, it does not mean that e-cigarettes were a cause because of overdetermination. It could be that the individual would have also started smoking in a world that lacked e-cigarettes, triggered by some other event. People’s testimony about the proximate trigger for smoking initiation can be reliable, but their assessment about whether the outcome was overdetermined will be much less reliable.

By contrast, someone’s testimony about conscious adoption of THR is quite reliable. Anyone who testifies that her history of product usage includes both smoking and a low-risk alternative, and that she intentionally switched from the former to the latter, is almost certainly correct. One simple survey question is sufficient to rule out a gateway effect in favor of reverse causation in such cases, and the failure to ask that question indicates a lack of serious interest in whether there really is a gateway.

Overdetermination is not a problem here. The overdetermination challenge does reemerge if we try to extend the causal statement to the common stronger claim “…and I would still be smoking were it not for THR” (though other details can help support this claim, and thus the testimonial evidence can still be compelling). But even if the smoker would have quit via other means, the testimony about the switching pattern is sufficient to rule out the gateway. 

Testimonial evidence is seldom collected in a manner that allows estimation of population-level statistics. But testimonials can rule out gateway cases, as noted, and can also identify *potential* true gateway cases, though overdetermination would prevent these from being sufficient evidence. It is noteworthy that those who claim that e-cigarettes are a gateway to smoking have failed, to my knowledge, to produce even a single testimonial in which someone reports being such a potential case.

### 2.6. Use of Population Prevalence Data is Not Promising

If use of low-risk tobacco products becomes popular in a population, then a theoretically testable prediction of the gateway claim is there will be more smoking. In the only population (Sweden) where a low-risk tobacco product (snus) has been sufficiently prevalent that such an effect could have be seen, Rodu *et al.* argued that there was no apparent gateway effect [20]. It is not possible to test this prediction during a transition period, where many people are switching from smoking to the alternative (either literally or via cohort replacement); under those circumstances, there could be a gateway effect, but it is masked by the stronger reverse causation trend. However, in Sweden the rate of male snus use stabilized at a high level while smoking rates remained low, which is contrary to the implications of a substantial gateway effect.

By contrast, recent similar claims that e-cigarettes cannot be causing a gateway because smoking continues to decline as e-cigarette use becomes more popular are fallacious. E-cigarette usage prevalence is low compared to smoking (e-cigarette trialing incidence rates among younger people appear to be similar to smoking in populations where e-cigarettes have become popular, but an actual usage practice appears to be remaining much lower). E-cigarette use is dominated by former smokers who intentionally used e-cigarettes to quit smoking, and thus are unlikely candidates for a gateway effect, and indeed are arguably not at risk of it (depending on the exact definition). Thus, when we consider the very few people who adopted e-cigarette use other than for THR, even if half of them were caused to start smoking (presumably far more than even gateway proponents suggest could happen), it would not be detectable. Any signal would be completely swamped by the trends in smoking prevalence and the noise in those estimates, to say nothing of the countervailing THR effect.

The gateway hypothesis could be amended to say that a low-risk alternative causes uptake of cigarettes in a population *where cigarettes are currently a lot more popular than the alternative*. This caveat might be seen as cynical expedience, but it is not unreasonable. The claim would be that when people start using any tobacco product, it causes some to drift among multiple tobacco products, and social forces will cause more to drift toward and settle on the most popular product. Indeed, this is the obvious explanation for observations that a larger portion of people who use smokeless tobacco in the USA equilibrate as smokers rather than the other way around, while the converse occurs in Sweden. With the addition of this nuance, no population prevalence data could ever effectively test the hypothesis: Once the alternative was sufficiently popular to extract the signal from the noise, smoking would be sufficiently unpopular that it nullified the claim.

Thus, while the Swedish data provides evidence against a simplistic gateway claim, no such data will be available for other products or other populations for the foreseeable future, and even then it would not rule out that there is still a gateway while smoking is far more popular.

### 2.7. Experimental Studies

Though not a realistic option, it is useful to consider the implications of a gateway experiment, where abstainers are assigned to use low-risk tobacco products to see if that causes smoking. The advantage of experiments (a.k.a. randomized controlled trials) is that they can eliminate systematic confounding and reverse causation as explanations, and are not affected by population trends. However, the differences between any operationalizable experiment and the real exposure of interest, inevitably introduced by an artificial controlled setting when studying complicated human behaviors, tend to be worse than the problems that are solved. It is often claimed that the reason that randomized trials of tobacco use behavior are not promising is that they would be unethical and impractical; while that is largely true, it is not actually the fundamental problem.

The conceptually ideal experiment, always useful to consider when contemplating research design, would be to run two histories of the world, one where the low-risk alternative existed and one where it did not. This would allow observations about how many nonsmokers in the latter world are smokers in the former (with the added bonus of observing how many smokers quit only because of THR). Monitoring corresponding individuals in the parallel worlds would solve the problem of merely analyzing aggregate counts, which shows only the net change from gateway and THR effects. Many would argue that from a practical standpoint only the net effect matters (though there are valid ethical objections to such netting), but it does not answer the specific question about a gateway.

The conceivable real-world experiment, which always falls far short of the theoretical ideal in social science, consists of selecting a random collection of nonusers of tobacco products, from the population (age group, *etc.*) of interest, and conscripting a random sample of them into using a low-risk alternative. If they became smokers more often than those who were not conscripted or were conscripted to a null-treatment arm, it would suggest there is a gateway effect. But conscripted use would obviously be quite different from real-world adoption so this experiment would tell us little.

Observational studies in epidemiology are typically misinterpreted as if they were an experiment, but the fundamental differences are obvious in this case. People who voluntarily try the low-risk products are not a random sample from all nonusers (*i.e*., there is important confounding), those who transition from trialing to using are not a random sample of them (further confounding), and many of them may be trying to substitute for their inclination to smoke, even those who are not currently smokers (reverse causation). Using the only available evidence of real-world behavior—which is observational—is much more difficult than interpreting results from an ideal experiment. 

### 2.8. Magnitude Matters

Before considering what available evidence would be useful to support or refute the hypothesis, it is important to quantify the hypothesis. If it is merely “there is one person who has been caused to start smoking due to use of the low-risk alternative” then there are no realistic statistics that could address the claim. A testimonial by an individual could support that claim, though the overdetermination problem would call the “I would have otherwise never started smoking” aspect of the testimony into serious doubt unless the individual is an older never-smoker. If the hypothesis is “half of would-be never-smokers who use the alternative product will become smokers,” available statistics, used properly, would address the claim. It would be easy to observe that more than half (to account for those destined to become smokers anyway) of never-smoker users of the alternative become smokers.

Presumably the hypothesis falls somewhere in between the former inconsequential claim and the latter implausible claim. “Presumably”, because proponents of gateway effect never quantify the claim they are making. This is a serious problem, both for the policy discourse and the epistemology; without quantification, the claim is disingenuous and research is meaningless. 

To make the hypothesis of interest both plausible and large enough to matter, it probably makes sense to test a claim in the order of 5% of would-be never-smokers who take up the alternative product are caused to smoke.

### 2.9. How Can Researchers Assess Whether There is a Gateway?

It is not entirely clear that any systematic study can detect gateway behavior of that magnitude, given how overwhelming the confounding is. If there is any hope, it needs to be based on thoughtful analysis about what constitutes evidence.

#### 2.9.1. The Need for Testimonial Evidence

Testimony-based research would be useful for directing quantitative analysis. Given that the proposed gateway behavior defies the welfare-maximizing logic described above, it is difficult to know what data to collect without some research about the nature, motives, and dynamics of apparent gateway cases. Naively hunting for evidence of a gateway effect in quantitative data is likely to be a dead-end, doing nothing to resolve competing hypotheses. But if the specifics of apparent gateway cases were documented, it might be possible to refine the statistical analysis.

For example, it might be that most purported examples of gateway cases would include a story like, “I tried a few packs of cigarettes a while ago, but did not really like them; but then I used e-cigarettes every day for six months and found myself thinking that a real cigarette sounded good after all.” If that were the case, the systematic research should focus on those who had previously consumed some quantity of cigarettes but had never become a smoker, and then later used e-cigarettes for a substantial period. It is also possible that such testimonials would reveal potential mediation analyses (statistical analyses based on intermediate variables that are a step along a causal pathway), with testable implications that would not be lost in the noise as the aggregate values are. If it were found that self-identified gateway cases mostly followed a particular pattern of smoking adoption that was generally not common among those starting smoking via other causal pathways, that pattern could be investigated in population statistics. This is a generalizable strategy when attempting to distinguish a weak causal relationship from stronger confounding effects.

Currently such hypotheticals are moot because it appears that no gateway proponent has ever offered the testimony-based *prima facie* evidence that there are *any* instances of gateway behavior. Statistics alone, absent case studies that suggest plausibility, cannot justify such an economically extraordinary claim. Without evidence of even a single case, the claim is not even a defensible hypothesis and is more like a religious myth or philosopher’s demon theory, something made up from whole cloth for rhetorical purposes.

#### 2.9.2. Bayesian Analysis Shows How to Avoid Garbage-In-Garbage-Out

Obvious Bayesian reasoning shows the lack of useful information in the recent studies of e-cigarettes. The observed statistics reduce one’s posterior probabilities that there is no association (which should have a very low prior in the first place), but do not change the relative probabilities of the competing explanations for the association. This means that gateway claims based on associations are examples of “faith-based Bayesianism”. 

To take an example I presented previously [17], consider someone whose prior beliefs are that there is a 50% chance that e-cigarettes cause smoking, a 1% chance that e-cigarettes are used for THR, a 1% chance that there is a strong association caused by confounding, with the remaining probability that there is no association. Further assume that there is an 80% probability a study will show an association under the first three hypotheses and a 5% chance under the last. (This characterization is not really proper since magnitude matters for updating the priors, not just this dichotomous characterization that is typical in epidemiology, but set that aside). The posterior probabilities after observing an association, applying the Bayes Theorem, would be 0.91, 0.02, 0.02, and 0.05, which appears to be strong support for the first hypothesis. But, of course, the observation increased the probabilities of each of the first three hypotheses by the same ratio, at the expense of the fourth, and so the overwhelming support for the first compared to the second and third is just an echo of the prior beliefs. This is not valid empirical reasoning, and represents the common critique of simplistic Bayesian analysis: garbage in, garbage out.

#### 2.9.3. Hypothetico-Deductive Reasoning

A good hypothetico-deductive test is based on asking, “what would we expect to observe if this hypothesis were true *which we would not expect to observe it were false*?” Such reasoning has recently been discussed in epidemiology under the rubric “negative controls for confounding.” An example of this is observing that the association of better health and getting an influenza vaccination in an elderly population is not stronger during flu season, which is not what we would expect if the association were caused by the vaccine working rather than by confounding [21]. Similarly, if an ostensible cause is strongly associated with an outcome variable that it cannot conceivably cause or be caused by, and this persists after “controlling for confounding”, it is apparent that there is uncontrolled residual confounding that may explain the main relationship of interest (as with the example of motorcycling being associated with smokeless tobacco use).

It is often possible to devise hypothetico-deductive tests that can be performed on a particular dataset. Unfortunately there is no easy recipe for identifying them, and they need to come from *ad hoc* scientific reasoning. An example of this appears below.

Another possible application of hypothetico-deductive reasoning is to take advantage of natural experiments. Where there is spatial heterogeneity of low-risk tobacco product use within a similar population, the gateway hypothesis predicts corresponding heterogeneity of smoking adoption rates. The popularity of e-cigarettes among teenagers appears to have involved substantial spatial heterogeneity across schools, due to contagion effects [22] (*i.e*., it is a social process that is either adopted in a social group or not). For years there were reports of some American high schools repeatedly confiscating e-cigarettes from students, while contemporaries at other schools reported never having seen one. Though overall smoking trends are uninformative for the reasons cited above, the gateway hypothesis makes both positive and negative prediction regarding this heterogeneity, which could provide a good test. Something similar might be possible among Norwegian users of smokeless tobacco, where there has been explosive adoption, unlike the relatively steady state in Sweden. Anyone genuinely interested in detecting a gateway effect should take advantage of this situation before the heterogeneity disappears. Indeed, it may already be too late to collect such data, but existing recent data might be available and new examples might be found as e-cigarettes become popular in countries where they are not yet.

#### 2.9.4. Propensity Scores: Mimicking the Ideal Experiment

The idealized experiment shows that we want to identify people who would not have been a smoker in a world that lacked the alternative product. The real world does not allow such definitive identification, but tools like propensity scoring or multi-stage regression statistically predict the probability someone would have become a smoker from available variables. With a sufficiently good predictor, we could notice if the ostensible gateway product was substantially changing the likelihood of someone smoking. Apparently only one study ever attempted this for THR products, and found no evidence of a gateway effect [23].

This approach is common in some social sciences, but is largely absent from substance-use epidemiology even though the concept of propensities is a natural fit there. Epidemiology’s use of arbitrary and largely untested covariates as “control” variables is an inadequate substitute for propensity scoring or other hypothesis-based statistical models, though it can still be sufficient to show that confounding may explain an uncontrolled result [24]. 

A proper propensity score is a reasonable predictor of the effect (smoking) using variables that are *mostly* independent of the ostensible cause (low-risk product use). The “controlling for confounding” approach could use the same demographic and personality covariates (neighborhood, career track, propensity for risk taking, *etc.*), but even for the best possible application of that approach, measurement error, simultaneity, and imperfect proxying for the true common causes will leave residual confounding that will inevitably create an association. By contrast, an effective propensity score eliminates simultaneity and the need for careful consideration of causal pathways, and though imperfect prediction means that the effect size estimate will be attenuated, any apparent effect will be better evidence the phenomenon exists. 

Ideally the variables used to create the propensity score are completely independent of the causal variable of interest. This is unlikely to be possible in the present context since the main problem is that of common causes. (As an aside, note that this means that the similar strategy, use of instrumental variables to deal with simultaneous causation, is not promising. This is largely moot, however, since the instrumental variables are particularly valuable when analyzing time series data. When examining cross-sectional data, there is little reason to use this method rather than a full-on two stage method.) However, this is not a fatal problem. So long as the variables used to measure propensity cannot plausibly be *caused by* low-risk product use and are not intermediate steps on a pathway by which low-risk product use causes smoking, the approach is still valid. The addition of temporal data can help determine the direction of causation.

In the case of e-cigarettes, fairly recent data from before e-cigarettes were available could be used to create the score, which eliminates the possibility that e-cigarette use affected any of the variables. A recurring theme here is that researchers interested in honest attempts to detect a gateway effect need to take advantage of a temporary window, before almost-random heterogeneity disappears and propensity data from before the ostensible gateway product became popular is too old. Once this window closes across the populations of interest, it is not clear there is much hope of sorting out the effects of competing causes until such a time that Swedish-style stability is reached.

### 2.10. A Checklist for Evaluating Empirical Research about Gateway Effects

To summarize the above, the following considerations should be applied to the design and interpretation of any empirical study of the gateway effect. Many of these principles generalize to other attempts to distinguish among the three possible causes of an association in cross-sectional data.

1. Is the research based on (a) a clear statement of what “gateway” means and (b) a hypothesized quantification of what is being tested or claimed? Failing either of those, the effort is only propaganda, not science. Is there any presentation of (c) a theory of why there would ever be gateway behavior or (d) testimonial evidence suggesting there is any such case and how it came about? Failing both of those, it is unlikely that the statistical analysis will be anything other than a misguided fishing expedition.

2. Does the result or potential result make any effort to discriminate between a gateway effect and causation in the other direction (THR) or confounding? If not, it is completely uninformative about whether a gateway, rather than just one of those established sources of association, is occurring.

3. Is the attempt to deal with the inevitable confounding based on some theory of the nature of the confounding (*i.e*., addressing the counterfactual or hypothetical experiment concept) and is it empirically tested (e.g., by checking the sensitivity to combinations of variables), or does it just consist of throwing in whatever covariates are conveniently available? If the latter, it is probably little better than no attempt to control for confounding, as is likely to be demonstrated by the persistence of negative control associations. If the former, the stated confidence in its success should still be epistemically modest.

4. Do the study results support any prediction that would be true under the hypothesis but unlikely if the hypothesis is false? If the data is highly consistent with the hypothesis being false, it obviously does not provide much support for the hypothesis.

5. Is there any attempt to detect temporal ordering of behaviors? As noted, this is neither necessary nor sufficient for a gateway, but the right ordering tends to better support the claim for obvious reasons. In particular, if the data shows that most of the association results from people who are already smokers trying the other product (as is likely for e-cigarettes and other THR products), and if it appears that the association is causal, then the causation is apparently flowing in the other direction.

6. Did the authors report enough different cuts at the data to make clear that any conclusion the data supports a particular hypothesis is apparently not an artifact of the specific models and results that are reported? Researchers can easily check how their results change based on innocuous-seeming details in their models (such as choosing among multiple candidate definitions of smoking, which covariates to control for, which cohorts from a dataset to use, how to categorize continuous variables, *etc.*) and choose the result they like best, leaving the readers (including journal reviewers) unable to detect this was done. This is an overarching problem with epidemiology, not specific to the gateway question [25]. But it is especially critical for highly politicized hunts for a tiny signal amidst a lot of noise. It is trivial to cherry-pick models and results that tend to support the author’s preferred claim. Adoption of methods that mitigate this is clearly beneficial (e.g., [26]).

### 2.11. An Application of These Principles

It is possible to apply these principles to a recent article by Dutra and Glantz (2014) [15] that was widely claimed to show there is a gateway effect. This included claims by the authors in various concurrent and subsequent communications [e.g., 27,28,29,30], which suggest this claim was their main interest, though they avoided the term “gateway” in the paper itself. (Details of their claims can be found in my previous analysis [17].) Doing so shows:

(a) The results completely failed to support what the authors and other commentators claimed, and
(b) the variables available in their dataset were really inadequate to answer the gateway question, however
(c) further analysis of the dataset shows that the data can be used more effectively, and that it shows the authors’ claim is even less well supported than the originally reported statistics implied.

The paper used the 2011 and 2012 U.S. National Youth Tobacco Survey to look at the association, among teenage students, between smoking and what they refer to as e-cigarette “use”. (This is a misleading term, since the survey only measures *trying* e-cigarettes and does not distinguish what we would think of as use.) The analysis was limited to subjects who reported having ever smoked, at least once, and the endpoints consisted of the available measures of smoking, specifically smoking a total of 100 cigarettes and having smoked (at all) in the last 30 days. Note that the original authors made odd use of the available data (e.g., omitting anyone who had never tried a cigarette; using at least one idiosyncratic definition) and only reported the results from a single model without explaining their choice. But the present analysis is not an attempt to critique their entire approach. In order to focus on the above-cited methodological points, what follows is based on replicating their approach—methods, models, and definitions—as closely as possible. This should not be seen as an endorsement of that approach.

The authors observed strong associations between measures of smoking and measures of e-cigarette trying (their Table 2 and Table 3), which serves as the entire basis for the gateway claims. A partial replication of their main results appears in Table 1 (note: all tables are in the Appendix). They claimed emphatically that their results were evidence that e-cigarettes were a gateway to smoking. 

Point (a) was presented in detail in my previous analysis. In particular, the original paper involved *no* attempt to discriminate between a gateway effect and either confounding (which the authors did not even seem to recognize exists; they never even used the word) or reverse causation. If the exposure and endpoint variables are reversed, the association remains, as will always be the case (this is illustrated in Table 2 for concreteness). If having tried an e-cigarette predicts being a smoker (the gateway phrasing), then being a smoker predicts having tried an e-cigarette (the THR phrasing). The results support no prediction that would be true under the gateway hypothesis that would not also be true if the hypothesis were false (unless the only alternative hypothesis considered is the implausible alternative that there is no association between using one tobacco product and using another). 

Moreover, the authors and others who made gateway claims about this data offered no theory of why or under what circumstance a gateway might occur, nor did they give any indication of what magnitude of gateway effect they were testing for or claim to have found. This makes clear that they were not making a serious attempt to empirically validate a scientific hypothesis.

Given the lack of acknowledgment of confounding in the original paper, it is no surprise that there was no serious attempt to control for confounding. Moreover (point (b)), the NYTS dataset is simply not rich enough to do so. The original authors controlled for age (which is obviously critical when studying a “have you ever…” phenomena), gender (also important for drug-use behavior), and race (which is rather less useful, though might be a very rough proxy for some cultural phenomena). They control for nothing else (other than which year of data collection a subject was in), and indeed, there is very little else they could have done with this data to assess propensity to use tobacco products. It should be obvious that those few variables do not come close to serving as good propensity estimators or proxies for underlying common causes. Thus there is simply no way this dataset can be used to discriminate a gateway effect from the inevitable confounding.

However, the data can be used more effectively than the original authors attempted. The original authors ignored data that would help predict, albeit roughly, which subjects were smokers before they ever tried e-cigarettes. The dataset is very limited, asking temporal questions only about cigarettes, but since e-cigarettes were a very new phenomenon at the time of the survey, it is safe to infer that almost everyone who tried e-cigarettes had first done so recently. 

Reiterating the point that temporality is not decisive (one way or the other) but is useful (since precedence is undoubtedly associated with causation), the data shows that of those defined as “current smokers” who had tried an e-cigarette, about three quarters (from the 2011 survey) or half (from the 2012 survey) tried their first cigarette before 2009 (Table 3). (This is based on their age at the time of the survey and their reported age of trying their first cigarette, as detailed in the Appendix; there is obviously imperfect correspondence between this and calendar year, but it is described in calendar terms for clarity.) This almost certainly means they tried a cigarette before trying an e-cigarette. Of course, many of those who first tried a cigarette later than 2009 also did so before trying an e-cigarette. While this cannot rule out gateway cases for this subpopulation (*trying* a cigarette does not immunize you from a later event causing you to become a *smoker*), it seems to provide better support for the reverse-causation THR claim than the gateway claim. A clear majority of the smokers who tried an e-cigarette tried a cigarette first, and we can surmise that most of them were smokers before trying an e-cigarette.

We can improve on this simplistic cut at the temporality data with a more formal hypothetico-deductive test. It makes use of the novelty of e-cigarettes as described above—*i.e.*, gateway cases would have to have started smoking fairly recently. (We could do much better if we had temporal data on when subjects first tried an e-cigarette.)

If the association of smoking and e-cigarette trialing or use is substantially explained by the gateway effect, then the association should increase as we narrow the population of smokers toward those who are more likely to be gateway cases. Those who more recently tried their first cigarette are more likely to be gateway cases because they are more likely to have become smokers recently. If many of them were caused to smoke by their (necessarily) recent e-cigarette use, the association will become stronger when we remove the dilution that results from including those who were more likely to have started smoking before they ever saw an e-cigarette. If there is a stronger association among the group that tried a cigarette earlier (in calendar time), and thus almost certainly has more established smokers, it would suggest that THR or some other reverse-causation is more likely because established smokers are the ones who are more often adopting e-cigarettes. If there is little difference, it will tend to support (though obviously not definitively) the hypothesis that confounding dominates both of those explanations. (It is worth noting the epistemically important fact that this test was devised and this paragraph was drafted before the data was analyzed.) 

The simple counts of when subjects first tried a cigarette show earlier smoking among those who had tried e-cigarettes as compared to those who never tried e-cigarettes, which by itself is enough to favor the THR interpretation over the gateway interpretation. Repeating the original methodology for subpopulations divided by when they first tried a cigarette confirms this (details in the Appendix). For parsimony the two waves of the survey are combined, with a covariate for survey wave. I recognize that odds ratios (ORs), which were used to follow the original analysis, are a terribly misleading statistic for comparing proportions. However, they are adequate for showing whether associations are stronger or weaker between different subpopulations, which is the present goal. 

For an endpoint of having ever smoked 100 cigarettes, the OR for ever trying an e-cigarette for those who tried their first cigarette before 2009 is 7.7, *versus* 5.2 for those who tried their first cigarette more recently (Table 4). These numbers should not be over-interpreted: The results are similar, though the contrast is weaker, for “current smokers”. We do not see a trend when we step the cutoff through 2007, 2009, and 2010 (Table 5 and Table 6), and the result does not always show up when we look at the “last 30 days” e-cigarettes exposure variable (Table 7, Table 8 and Table 9). Still, this does show that the available data could be used to test a prediction of the hypothesis in question, and when that is done, the results provide somewhat better support for the THR explanation than they do the gateway hypothesis. The association between smoking and trying an e-cigarette is stronger when we select for long-term smokers and weaker as we select for those who might actually be gateway cases. 

This analysis was easy to perform, and recognizing its potential value in answering the scientific question took only a bit of serious contemplation. Its absence from the original authors’ analysis, along with the more blatant omissions already noted, suggest that the authors were seeking to support the conclusion rather than test the hypothesis. Moreover, journal editors and reviewers still signed off on publishing the paper, demonstrating a lack of critical analysis on their parts. 

In addition, this analysis illustrates the ease of cherrypicking results that support a particular claim when authors report only one of many potential results. The results, all considered, still support the conclusion that THR better explains the association than does the gateway claim. But had I followed the common practice in epidemiology and public health, reporting only the “best” numerical results (the left side of Table 4 or Table 5 or Table 7), it would have misled the reader into believing the support for the conclusion is stronger than it really is. Instead, this truth-seeking method of reporting of the results, showing the results for the other cuts at the data that occurred to me to check, shows that the support for that conclusion is rather weaker than the cherrypicked strongest numbers would have shown. A typical health journal article would not have even mentioned that the other calculations had been done, implying that the “best” model was the only one ever considered by the authors. 

It seems likely that such hints of support are the best we are likely to get on this topic, pointing out the need for careful methods, including sufficient reporting to show that authors are not cherrypicking much “clearer” results than the data really supports.

## 3. Conclusions

Searching for some signal of a gateway effect amidst overwhelming confounding and reverse causation requires more rigorous methods than are typical in public health epidemiology. This generalizes to any attempt to use cross-sectional data to sort out causation in a particular direction from confounding or reverse causation. When seeking epidemiologic associations where confounding is minimal or relatively simple in its causes, the typical methods used in the field are still far from optimal, but the empirical results might still be basically useful. That is not the case in this context. While it might never be possible to convincingly demonstrate a gateway effect given the challenges, and statistical analyses have no hope of detecting a tiny effect, there are clearly better and worse ways to pursue the question.

## Appendix

See the main text for context and how these results were used. The first table replicates the 2011 survey wave results from Table 3 of Dutra and Glantz (2014) [15]. The numbers are not quite identical because, as is typical for public health research, the methodology was not reported sufficiently to permit exact replication, but they are close enough to show that the present methodology replicates that from the original paper. As noted in the text, the original authors made various dubious modeling choices, but this is not an attempt to broadly critique their paper. Replicating, to the extent possible, their methodology is sufficient to make the points at hand.

The original authors defined “current smoking” to mean ever smoked 100 cigarettes and smoked (at all) in the last 30 days. The two e-cigarette variables are based on ever trying or using an e-cigarette (at all) and trying or using an e-cigarette (at all) in the last 30 days.

Many authors, including Dutra and Glantz, refer to these as “use” and “current use.” This is controversial, despite its parallel with the traditional characterization of smoking status, and has been used in political rhetoric to intentionally exaggerate e-cigarette use among teens. Any use in the last 30 days is a decent proxy for a current practice of smoking in an adult, since smoking tends to either be an established habit or entirely absent. But this is not so accurate for teenagers and especially for e-cigarettes, which are a novelty that teenagers might try in social settings (and since someone is unlikely to give away their e-cigarette, in contrast with the norm of giving a whole cigarette, so it could easily just be a single puff), or that smokers might use as an occasional convenient substitute. The “use” statistics are presented without such caveats, and the typical reader is likely to interpret “uses” to mean something like “many times per day, every day” even though an affirmative survey answer might refer only to taking a single puff ever, which happened to have been within 30 days.

Following the original, the estimated associations are presented as odds ratios (ORs), which are not the proper statistics to use when the endpoint is a proportion, but adequate for all present purposes.

**Table 1:** Partial replication of main Dutra-Glantz result: Association of smoking and e-cigarette trialing.

	Ever Smoked at Least 100 Cigarettes OR [95% CI]	Current Smoking OR [95% CI]
Ever tried e-cigarette	7.7 [5.5, 10.8]	
Recently tried e-cigarette		6.8 [3.9, 11.8]
Age	1.3 [1.2, 1.4]	1.3 [1.2, 1.4]
Black (ref = White)	0.36 [0.2, 0.6]	0.36 [0.2, 0.5]
Other race	0.72 [0.5, 1.0]	0.69 [0.5, 0.9]
Male	1.4 [1.1, 1.7]	1.4 [1.2, 1.8]

The next table shows the obvious result that the association remains if you reverse the ostensible cause and the ostensible effect (*i.e*., the dependent variable and the independent variable of interest).

**Table 2:** Association from Table 1, with dependent and main independent variables reversed.

	Ever Tried an E-Cigarette OR [95% CI]	Recently Tried an E-Cigarette OR [95% CI]
Ever smoked at least 100 cigarettes	7.7 [5.5, 10.8]	
Current Smoking		6.7 [3.9, 11.5]
Age	0.99 [0.9, 1.1]	0.84 [0.8, 0.9]
Black (ref = White)	0.40 [0.2, 0.7]	0.59 [0.3, 1.3]
Other	0.67 [0.5, 0.9]	0.76 [0.5, 1.2]
Male	1.3 [1.0, 1.7]	2.3 [1.5, 3.6]

The following counts identify the portion of the “current smokers” study population that tried their first cigarette more and less recently. Note that the description of this is simplified to “before 2009” *vs.* not, because calendar time is important for the analysis, based on the emerging availability of e-cigarettes. The survey, however, did not ask about calendar time of first trying, but age at first trying. This along with current age of the subject allows a rough calculation of calendar time, but obviously the step functions do not line up perfectly. The definition of “before 2009” is {if [current age] − [age at first trying] >3}, for data collected in 2012, using >2 for data collected in 2011. Someone who is 18 on a given day in 2012 and tried a first cigarette at 14 (defined as “before 2009”) probably did so in 2008 (assuming every distribution in sight is close to flat), but it might have been 2007 or 2009.

**Table 3:** Time of trying first cigarette for current smokers, by e-cigarette trialing status.

Year Tried First Cigarette	Never Tried E-Cigarette	Ever Tried E-Cigarette
**2011 Survey Wave**		
before 2009	51% (*n* = 788)	71 (229)
2009–2011	49 (750)	29 (94)
**2012 Survey Wave**		
before 2009	33 (412)	44 (386)
2009–2012	67 (835)	56 (486)

The following table follows the original methodology, as described above, with the exception of pooling the two survey waves and adding a covariate for which wave included the record. The population is divided into those who first tried a cigarette before 2009 *versus* more recently, as well as can be calculated per the above. The purpose of this analysis is explained in the main text.

**Table 4:** Association of smoking and e-cigarette ever trying, by time of first trying a cigarette: before 2009 *vs.* later (ORs and 95% CIs).

	Dep. Variable = Ever Smoked			Dep. Variable = Current Smoker		
	All	Before 2009	2009 and After	All	Before 2009	2009 and After
Ever tried e-cigarette	6.5	7.7	5.2	6.3	6.7	5.2
	[5.4, 7.9]	[6.0, 9.8]	[4.1, 6.6]	[5.2, 7.5]	[5.3, 8.4]	[4.0, 6.7]
Male	1.3	1.3	1.2	1.3	1.3	1.2
	[1.2, 1.4]	[1.2, 1.4]	[1.1, 1.3]	[1.2, 1.4]	[1.2, 1.4]	[1.1, 1.3]
Age	1.4	1.4	1.2	1.4	1.4	1.3
	[1.2, 1.6]	[1.2, 1.7]	[1.0, 1.6]	[1.2, 1.6]	[1.1, 1.6]	[1.0, 1.6]
Black (ref = White)	0.38	0.28	0.36	0.43	0.35	0.36
	[0.3, 0.5]	[0.2, 0.4]	[0.2, 0.6]	[0.3, 0.6]	[0.2, 0.5]	[0.2, 0.6]
Other race	0.72	0.53	0.73	0.76	0.61	0.73
	[0.6, 0.9]	[0.4, 0.7]	[0.6, 0.9]	[0.6, 0.9]	[0.5, 0.8]	[0.6, 0.9]
Year 2012	0.58	0.64	0.89	0.58	0.63	0.87
	[0.5, 0.7]	[0.5, 0.8]	[0.7, 1.2]	[0.5, 0.7]	[0.5, 0.8]	[0.6, 1.2]
Observations	11,183	4396	6787	11,183	4396	6787

The following two tables repeat the previous table’s approach, substituting “before 2007” and “before 2010” for “before 2009” (the former two being defined analogously to the latter).

**Table 5:** Association of smoking and e-cigarette ever trying, by time of first trying a cigarette: before 2007 *vs.* later (ORs and 95% CIs).

	Dep. Variable = Ever Smoked			Dep. Variable = Current Smoker		
	All	Before 2007	2007 and After	All	Before 2007	2007 and After
Ever tried e-cigarette	6.5	8.7	6.0	6.3	6.9	6.0
	[5.4, 7.9]	[6.4, 11.7]	[4.9, 7.4]	[5.2, 7.5]	[5.2, 9.1]	[4.8, 7.4]
Male	1.3	1.3	1.2	1.3	1.2	1.3
	[1.2, 1.4]	[1.2, 1.4]	[1.2, 1.3]	[1.2, 1.4]	[1.1, 1.3]	[1.2, 1.3]
Age	1.4	1.6	1.3	1.4	1.6	1.3
	[1.2, 1.6]	[1.3, 2.1]	[1.1, 1.5]	[1.2, 1.6]	[1.3, 2.1]	[1.1, 1.5]
Black (ref = White)	0.38	0.25	0.37	0.43	0.35	0.39
	[0.3, 0.5]	[0.2, 0.4]	[0.2, 0.6]	[0.3, 0.6]	[0.2, 0.5]	[0.3, 0.6]
Other race	0.72	0.63	0.62	0.76	0.81	0.60
	[0.6, 0.9]	[0.5, 0.8]	[0.5, 0.7]	[0.6, 0.9]	[0.6, 1.1]	[0.5, 0.7]
Year 2012	0.58	0.57	0.69	0.58	0.56	0.69
	[0.5, 0.7]	[0.4, 0.8]	[0.5, 0.9]	[0.5, 0.7]	[0.4, 0.7]	[0.5, 0.9]
Observations	11,183	2281	8902	11,183	2281	8902

**Table 6:** Association of smoking and e-cigarette ever trying, by time of first trying a cigarette: before 2010 *vs.* later (ORs and 95% CIs).

	Dep. Variable = Ever Smoked			Dep. Variable = Current Smoker		
	All	Before 2010	2010 and After	All	Before 2010	2010 and After
Ever tried e-cigarette	6.5	6.6	5.5	6.3	6.0	5.4
	[5.4, 7.9]	[5.3, 8.3]	[4.2, 7.1]	[5.2, 7.5]	[4.9, 7.4]	[4.1, 7.2]
Male	1.3	1.3	1.1	1.3	1.3	1.1
	[1.2, 1.4]	[1.3, 1.4]	[1.0, 1.2]	[1.2, 1.4]	[1.2, 1.4]	[1.0,1.2]
Age	1.4	1.4	1.3	1.4	1.4	1.4
	[1.2, 1.6]	[1.2, 1.7]	[1.0, 1.8]	[1.2, 1.6]	[1.1, 1.6]	[1.0, 1.8]
Black (ref = White)	0.38	0.30	0.37	0.43	0.36	0.36
	[0.3, 0.5]	[0.2, 0.4]	[0.2, 0.6]	[0.3, 0.6]	[0.3, 0.5]	[0.2, 0.7]
Other	0.72	0.57	0.81	0.76	0.63	0.80
	[0.6, 0.9]	[0.5, 0.7]	[0.6, 1.1]	[0.6, 0.9]	[0.5, 0.8]	[0.6, 1.1]
Year 2012	0.58	0.69	1.1	0.58	0.68	1.0
	[0.5, 0.7]	[0.5, 0.9]	[0.7, 1.6]	[0.5, 0.7]	[0.5, 0.9]	[0.7, 1.5]
Observations	11,183	6040	5143	11,183	6040	5143

The following three tables repeat the above three tables, for recently trying or using an e-cigarette rather than ever trying.

**Table 7:** Association of smoking and e-cigarette recent trying, by time of first trying a cigarette: before 2009 *vs.* later (ORs and 95% CIs).

	Dep. Variable = Ever Smoked			Dep. Variable = Current Smoker		
	All	Before 2009	2009 and After	All	Before 2009	2009 and After
Recently tried e-cigarette	7.5	7.2	7.4	7.7	7.4	7.8
	[5.6, 10.0]	[5.1, 10.3]	[5.1, 10.6]	[5.8, 10.3]	[5.4, 10.1]	[5.3, 10.9]
Male	1.3	1.3	1.2	1.3	1.3	1.2
	[1.3, 1.4]	[1.3, 1.4]	[1.1, 1.3]	[1.3, 1.4]	[1.2, 1.4]	[1.1, 1.3]
Age	1.4	1.4	1.3	1.4	1.4	1.3
	[1.3, 1.7]	[1.2, 1.7]	[1.0, 1.6]	[1.2, 1.6]	[1.1, 1.6]	[1.0, 1.7]
Black (ref = White)	0.30	0.21	0.31	0.34	0.26	0.31
	[0.2, 0.4]	[0.1, 0.3]	[0.2, 0.5]	[0.3, 0.4]	[0.2, 0.4]	[0.2, 0.5]
Other	0.64	0.46	0.68	0.67	0.51	0.67
	[0.5, 0.8]	[0.4, 0.6]	[0.5, 0.9]	[0.6, 0.8]	[0.4, 0.6]	[0.5, 0.9]
Year 2012	0.76	0.82	1.1	0.74	0.79	1.1
	[0.6, 0.9]	[0.7, 1.0]	[0.9, 1.5]	[0.6, 0.9]	[0.6, 1.0]	[0.8, 1.5]
Observations	11,183	4396	6787	11,183	4396	6787

**Table 8:** Association of smoking and e-cigarette recent trying, by time of first trying a cigarette: before 2007 *vs.* later (ORs and 95% CIs).

	Dep. Variable = Ever Smoked			Dep. Variable = Current Smoker		
	All	Before 2007	2007 and After	All	Before 2007	2007 and After
Recently tried e-cigarette	7.5	9.7	6.6	7.7	9.0	6.9
	[5.6, 10.0]	[5.8, 16.2]	[4.7, 9.1]	[5.8, 10.3]	[5.7, 14.3]	[5.0, 9.6]
Male	1.3	1.3	1.3	1.3	1.3	1.3
	[1.3, 1.4]	[1.2, 1.4]	[1.2, 1.4]	[1.3, 1.4]	[1.2, 1.4]	[1.2, 1.4]
Age	1.4	1.6	1.3	1.4	1.6	1.3
	[1.3, 1.7]	[1.3, 2.0]	[1.1, 1.6]	[1.2, 1.6]	[1.2, 2.0]	[1.1, 1.6]
Black (ref = White)	0.30	0.19	0.30	0.34	0.26	0.32
	[0.2, 0.4]	[0.1, 0.3]	[0.2, 0.5]	[0.3, 0.4]	[0.2, 0.4]	[0.2, 0.5]
Other	0.64	0.53	0.56	0.67	0.66	0.54
	[0.5, 0.8]	[0.4, 0.7]	[0.5, 0.7]	[0.6, 0.8]	[0.5, 0.9]	[0.4, 0.7]
Year 2012	0.76	0.72	0.91	0.74	0.67	0.90
	[0.6, 0.9]	[0.5, 1.0]	[0.7, 1.2]	[0.6, 0.9]	[0.5, 0.9]	[0.7, 1.2]
Observations	11,183	2281	8902	11,183	2281	8902

**Table 9:** Association of smoking and e-cigarette recent trying, by time of first trying a cigarette: before 2010 *vs.* later (ORs and 95% CIs).

	Dep. Variable = Ever Smoked			Dep. Variable = Current Smoker		
	All	Before 2010	2010 and After	All	Before 2010	2010 and After
Recently tried e-cigarette	7.5	7.2	7.2	7.7	7.4	7.3
	[5.6, 10.0]	[5.1, 10.3]	[4.9, 10.6]	[5.8, 10.3]	[5.3, 10.4]	[4.9, 10.7]
Male	1.3	1.3	1.1	1.3	1.3	1.1
	[1.3, 1.4]	[1.3, 1.4]	[1.0, 1.2]	[1.3, 1.4]	[1.3, 1.4]	[1.0, 1.2]
Age	1.4	1.4	1.4	1.4	1.4	1.4
	[1.3, 1.7]	[1.2,1.6]	[1.0, 1.8]	[1.2, 1.6]	[1.2, 1.6]	[1.0, 1.9]
Black (ref = White)	0.30	0.23	0.30	0.34	0.28	0.29
	[0.2, 0.4]	[0.2, 0.3]	[0.2, 0.5]	[0.3, 0.4]	[0.2, 0.4]	[0.2, 0.5]
Other	0.64	0.49	0.74	0.67	0.54	0.74
	[0.5, 0.8]	[0.4,0.6]	[0.6, 1.0]	[0.6, 0.8]	[0.4, 0.7]	[0.5, 1.0]
Year 2012	0.76	0.88	1.4	0.74	0.85	1.3
	[0.6, 0.9]	[0.7,1.1]	[1.0, 2.0]	[0.6, 0.9]	[0.7, 1.1]	[0.9, 1.9]
Observations	11,183	6040	5143	11,183	6040	5143
